# Therapeutically Induced Modulation of Collagen I-to-III Ratio Three Weeks After Rabbit Achilles Tendon Full Transection

**DOI:** 10.3390/biology15020204

**Published:** 2026-01-22

**Authors:** Gabriella Meier Bürgisser, Olivera Evrova, Pietro Giovanoli, Maurizio Calcagni, Johanna Buschmann

**Affiliations:** Department of Plastic Surgery and Hand Surgery, University Hospital Zurich, 8091 Zurich, Switzerland; gabriella.meierbuergisser@usz.ch (G.M.B.); olivera.evrova@gmail.com (O.E.); pietro.giovanoli@usz.ch (P.G.); maurizio.calcagni@usz.ch (M.C.)

**Keywords:** Herovici, collagen I, collagen III, immunohistochemistry, mature collagen, young collagen, full transection model, rabbit, Achilles tendon, tenotomies

## Abstract

During tendon healing, collagen III expression precedes that of collagen I. The collagen I-to-III ratio at a certain time point post-laceration serves as an indicator of the healing status. Consequently, it is crucial to understand how different therapeutic approaches to support tendon healing affect the collagen I-to-III ratio in the extracellular matrix of a healing tendon, particularly across distinct anatomical zones. We compared the impact of a platelet-derived growth factor-BB (PDGF-BB) treatment via controlled release from coaxially electrospun DegraPol^®^ (Ab medica, Cerro Maggiore, Italy) hollow-fiber mesh with a treatment by the vehicle alone (no PDGF-BB) in the rabbit Achilles tendon full transection model and provide data on the collagen I-to-III ratio 3 weeks post-operation. For this purpose, we compared a dual-color Herovici staining to two single IHC labeling, for collagen I and collagen III, respectively. Herovici staining (HV) was expected to offer a more precise approach (pink-to-blue histogram) than the two separately labeled IHC stainings, both with chromogenic DAB labeling (red-to-green histogram), despite an anticipated positive correlation of the data assessed by these methods. Different zones were compared, i.e., native tendon tissue, reactive zone at interface to implant, hot zone within the core of the healing tendon and the zone within the scaffold, meaning the collagen deposited within the fibers of the implanted DegraPol^®^ tube, respectively. The analysis revealed that the ratios obtained via HV correlated weakly with the ratios obtained by IHC. Based on HV, PDGF-BB therapy led to higher collagen I-to-III ratios in all zones, except for the zone within the scaffold pores, while IHC did not reveal significant differences. Notably, collagen I-to-III ratios were not higher in immediate proximity, but rather distal from the PDGF-BB releasing implant, specifically in the core of the healing tendon tissue. Hence, a PDGF-BB therapy is suggestive of greater collagen maturation in specific zones of the healing tendon.

## Simple Summary

Achilles tendon ruptures, frequently occurring in both athletes and the elderly, are characterized by a slow healing process. To accelerate repair, novel implant materials have been developed. We evaluated an implant designed to release a specific growth factor, using a rabbit Achilles tendon full-transection model, with the material placed around a conventional suture. To monitor healing progression, we assessed the collagen I-to-III ratio, as collagen III is typically deposited first and later replaced by collagen I. This study compared a growth factor-releasing implant against an empty control. In particular, we analyzed different local zones of the healing tendon. Additionally, we compared two staining methods for measuring the collagen ratio: a cost-effective, simultaneous staining method (Herovici) versus a more expensive dual-labeling approach (Immunohistochemistry). Although we found a weak correlation between the two methods, the Herovici method was more discriminative in this specific model, allowing for a better assessment of collagen I-to-III ratios across different zones of the healing tendon. Based on Herovici, there were higher collagen I-to-III ratios for the healing zone in the core of the tendon, as well as for the reactive zone near the implant for the platelet-derived growth factor-BB (PDGF-BB) therapy, compared to the empty control. In addition, the ratio was higher in the healing tendon zone compared to the zone within the implant pores.

## 1. Introduction

Acute tendon injuries may adversely impact the patient, even after prolonged healing periods of up to a year. Because tendon tissue is highly avascular and tendon cells have low metabolic activity [1], the healing process is tedious and slow [2]. Frequently, scar formation and fibrotic adhesion to the surrounding tissues are observed, resulting in lower mechanical strength, leading to an increase in the patient’s risk for tendon re-rupture and an impaired and restricted range of motion. Thus, implants supporting the surgical repair of tendons are needed [3]. There are different research approaches to how such implant materials should be constructed, composed and made functional by delivering bioactive cues to the healing tendon. Examples include bioactive hydrogels with gradients of bioactive glass [4], a sustained release of TGF-β3 from porous titanium [5] or growth and differentiation factor-5 (GDF-5) incorporated into a polyurethane scaffold to support tendon healing [6]. Also, the beneficial effects of platelet-derived growth factor (PDGF) on tendon healing were reported, as PDGF stimulates proliferation and collagen synthesis [7,8].

Previously, we focused on the effects of growth factors released from electrospun DegraPol^®^ tubes that were set around fully transected and sutured rabbit Achilles tendons (ATs). These bioactive tubes resulted in lower adhesion formation for a combination of insulin-like growth factor-1 (IGF-1) and PDGF-BB [9]; and in twice-as-strong tendons 3 weeks post-operation, in case PDGF-BB was delivered to the ATs [10]. The promising results obtained from a PDGF-BB delivery, with respect to substantially increased biomechanical properties, were assumed to be caused, at least in part, by a higher collagen I expression in the extracellular matrix, indicating a more advanced healing state. During the three-stage healing cascade [2], first, collagen III is deposited, and only after 4 to 6 weeks is it replaced by collagen I [2]. Collagen I is the mature and mechanically stronger collagen compared to the premature weaker collagen III [11]. Thus, besides the stage-dependent cell and vessel density, the dynamics of this biological process enables the researcher to assess the ratio of collagen I-to-III as a proxy for the tendon healing status, with higher collagen I-to-III ratios representing a more advanced mature status. There is a research gap for cost-effective staining methods offering high accuracy and a broad dynamic range in tendon research. Moreover, zone-specific analysis of the collagen I-to-III ratio has not been performed in an acute rabbit Achilles tendon injury model so far.

Consequently, we assessed the collagen I-to-III ratio in different zones of a rabbit Achilles tendon after full transection, suture and coaxially electrospun DegraPol^®^ tube releasing PDGF-BB from the core of the fibers [10] three weeks post-operation and compared it to the same experiment with pure DegraPol^®^ tubes (not-treated contralateral specimen served as controls) for specific anatomical zones in the healing tendon. The main aim of the study was to discover differential impacts of PDGF-BB in different zones. In addition, we determined the collagen I-to-III ratio with two different methods: with dual color staining by Herovici (HV) and with immunohistochemical labeling (IHC) for the collagen I and III proteins, respectively, to reach a decision on which method might be better for this specific purpose in this specific model.

The hypotheses were as follows:

1. Consistency of the two methods: HV and IHC show similar collagen I-to-III ratios for different local zones in the healing tendon tissue.
2. PDGF-BB treatment efficacy: PDGF-BB releasing tube-treated tendons exhibit higher collagen I-to-III ratios in all zones compared to tube-only-treated tendons, with the magnitude of this increase varying across different anatomical zones of the healing tendon.
3. Spatial heterogeneity: Tendons treated with a PDGF-BB tube show the most pronounced collagen I-to-III increase within the scaffold pores and immediately adjacent to the scaffolds, compared to distal regions like the tendon core.

## 2. Materials and Methods

### 2.1. Preparation of DP Polymer Solutions and Incorporation of PDGF-BB in Polymer Solutions

DP polymer solutions of 10 wt% for coaxial electrospinning were prepared by dissolving the polymer overnight at room temperature in a mixture of chloroform/HFP (80:20 wt/wt) (Sigma-Aldrich, Buchs, Switzerland). For PDGF-BB incorporation into scaffolds via coaxial electrospinning, 100 μL of the PDGF-BB stock solution, containing a total of 8 µg PDGF-BB for bioactive DP tubes for in vivo experiments (based on previous dose response experiments [12]) were added drop-wise to 3.5 g of 30 wt% polyethylene glycol (PEG, 35 kDa) (Sigma-Aldrich, Buchs, Switzerland) polymer solution in MilliQ water (Sigma-Aldrich, Buchs, Switzerland), under stirring at 500 rpm for 15 min. Immediately afterwards, the solution was used for electrospinning [13].

### 2.2. Scaffold Production Coaxial Electrospinning

Bioactive core–shell DP scaffolds, composed of DP shells and PEG + PDGF-BB cores, were prepared by coaxial electrospinning. Additionally, 10 wt% DP was used as a shell polymer solution, while 30 wt% PEG solution with PDGF-BB, dissolved in MilliQ water, was used as a core polymer solution. The PEG was used to facilitate PDGF-BB dissolution in water. A spinneret with inner and outer needle diameters of 1 mm and 2.8 mm, respectively, was used. The polymer solutions for the shell and core were delivered separately, using two syringe pumps (SP210cZ and Aladdin-1000, WPI, Friedberg, Germany) and different flow rates (shell: 1.5 mL h^−1^; core: 0.5 mL h^−1^). A voltage of 17 kV was applied and the working distance between the spinneret and the collector was 18 cm. The process was performed at room temperature (22–24 °C) and at less than 35% humidity [10].

### 2.3. Bioactive DP Tubes Production by Coaxial Electrospinning

For bioactive DP tube production via coaxial electrospinning, a cylindrical rotating aluminum mandrel (length: 200 mm, diameter: 4 mm) was used as a collector. Pure 12 wt% DP polymer solutions were used for the outer layer of the DP tube, produced by single electrospinning (11–12.5 kV, 1 mL h^−1^ flow rate and 20 cm working distance).

The bioactive layer of the coaxially electrospun DP tubes was produced with 10 wt% DP solution as a shell and 30 wt% PEG + PDGF-BB polymer solution as a core. For the shell and core solutions, 1.5 mL h^−1^ flow rate and 0.5 mL h^−1^ flow rates were used, respectively, under 17 kV applied voltage and 18 cm working distance. The production was at room temperature and at less than 35% humidity. Electrospun tubes were removed from the collector and dried in a vacuum at room temperature [10].

### 2.4. In Vivo Implantation of the DP Tubes

For the in vivo study, 6 female New Zealand White rabbits, aged 12 to 16 weeks, that were specific pathogen-free (SPF) were used (Charles River, Research Models and Services, Germany). All animals were housed, maintained and fed as described earlier [10]. Ethical approval for the experiments was obtained from the veterinary office of Zurich, Switzerland (reference numbers 92/2009 and 193/2012). Prior to surgery, all animals were acclimatized to their environment for 2 weeks.

Before implantation, the DP tubes were sterilized with H_2_O_2_ (plasma sterilization). The full transection of the Achilles tendon 2 cm above the calcaneus, followed by a 4-strand Becker suture, was performed as previously described [14]. The DP tube was then flipped over the wound. Subsequently, the wound was closed with a running suture (using a USP 6.0 polypropylene fiber from B. Braun Medical AG, Sempach, Switzerland) of the fascia and interrupted skin. Immediately post-surgery, a Durogesic Matrix patch (Janssen-Cilag AG, Schaffhausen, Switzerland) was applied with 4.2 mg Fentanyl per patch to provide analgesia for about 72 h with 25 µg/h Fentanyl. The post-operative treatment included a cast with an angle of 180° at the ankle [15]. The cast was well padded [15]. Three weeks post-surgery, the rabbits were euthanized in deep anesthesia (100 mg/kg Ketamine and 4 mg/kg Xylazine) with 80 mg/kg Pentobarbital (Esconarkon ad us. vet., Uznach, Switzerland) and the tendons were extracted [16].

The 6 rabbits were randomly distributed into 2 groups (receiving coaxially electrospun DP tube without and with PDGF-BB), with 3 rabbits in each group. All rabbits were operated upon on one hind leg. The counter hind leg of all operated animals were not treated (NT) and served as controls.

### 2.5. Herovici Staining and IHC Labeling

For histological processing, 4%-formalin-fixed tendon tissue (24 h fixation) was embedded in paraffin blocks and sectioned into 5 µm slices.

For Herovici staining, a standard kit was used (18432; Morphisto, Offenbach am Main, Germany), yielding a pink color for collagen I and a blue color for collagen III. For IHC, an antigen retrieval (AR) step was performed, using 10 mM citrate buffer (pH 6.0) with 0.05% Tween-20 for 20 min at 95 °C. After that, the sections were blocked in 5% donkey serum and 1% bovine serum albumin (BSA) in 1× Tris Buffered Saline (TBS) for 1 h (at room temperature (RT)). Next, sections were incubated with a mouse monoclonal anti-collagen I antibody (ab90395; Abcam, Lucerne, Switzerland, 1:200 dilution) or a mouse monoclonal anti-collagen III antibody (AF5810; Acris, Wettingen, Switzerland, 1:200 dilution).

Dilutions of antibodies were either taken from the technical sheet data provided by the suppliers or by the titration of different concentrations and a comparison of staining intensities.

Chromogenic IHC was performed for collagen I and III, respectively. To this end, samples were blocked with 3% hydrogen peroxide solution in water for 10 min (RT) and subsequently washed 3 times with 1 × TBS. Blocking endogenous peroxidase is necessary for a peroxidase-based staining system to prevent false-positive staining reactions. The primary antibody detection was made with a biotinylated anti-mouse IgG secondary antibody and streptavidin–horseradish peroxidase (HRP) (ZytoChem Plus HRP Kit Mouse; Zytomed Systems, Muttenz, Switzerland). Then, colorimetric detection was conducted according to the manufacturer’s protocol with DAB (DAB Substrate Kit High Contrast; Zytomed Systems, Bargteheide, Germany). Last, slides were washed in tap water and mounted, using Faramount Aqueous Mounting Medium from Agilent Technologies, Basel, Switzerland.

With a slide scanner (Pannoramic 250 Flash II, 3Dhistech, Budapest, Hungary), images of whole tissue sections were taken. We made snapshots of fields of view (FOVs) with CaseViewer Software v.2.1 or imaged them with a Leica 6000 light microscope (Leica, Basel, Switzerland).

### 2.6. Quantification of Collagen I-to-III Ratios, Statistics and Data Presentation

Color intensities were measured quantitatively, using histogram analysis of Image J version 1.53e. For ECM labeling with a chromogenic detection system, red-to-green ratios were calculated. The collagen I-to-III ratio was assessed as the red-to-blue ratio in the histograms of the HV-stained sections, and as the red-to-green ratio for collagen I and collagen III IHC sections, whereby these ratios were divided to obtain the collagen I-to-III ratios for IHC labeling, according to Equation (1).

(1) $$Collagen\;I\text{-}to\text{-}III\;ratio\;for\;IHC=\frac{Red\text{-}to\text{-}green\;ratio\;in\;collagen\;I\;labelled\;section}{Red\text{-}to\text{-}green\;ratio\;in\;collagen\;III\;labelled\;section}$$

**Scheme 1:**
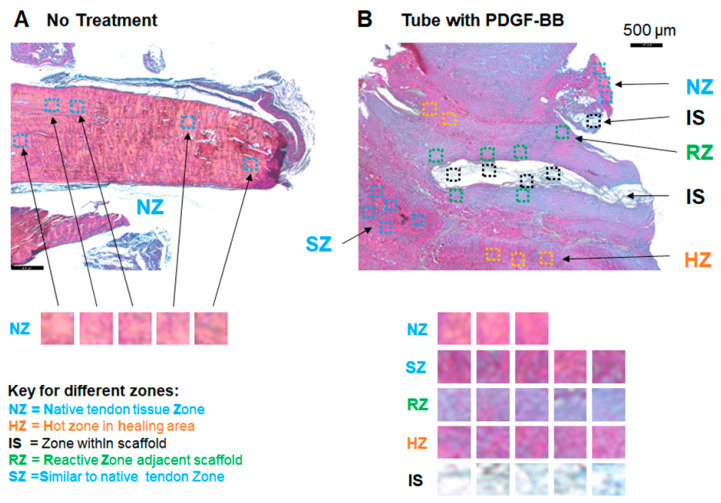
Illustration where the different zones are found in the histological sections. On the left, a no-treatment specimen is shown with 5 FOVs for NZ = native zone (**A**). On the right, a section of the experimental group tube with PDGF-BB is shown (**B**), and the different zones are exemplified and explained below. Both examples were stained with Herovici.

Different zones (Scheme 1) were assessed, and in each zone, 5 fields of view (FOVs) were analyzed (please see the Supporting Information for the FOVs). To present the data distribution of the collagen I-to-III ratios, Microsoft 365 Excel version 2506 was used, and linear regression analysis provided by this software was utilized to calculate the linear regression parameters (slope, intercept and correlation coefficient R^2^). For quantitative inter-group comparison, a Shapiro–Wilk test was used to test for the normal distribution of the data. For comparison of the two groups, an unpaired *t*-test was used in the case of the normal distribution of data. Otherwise, a Mann–Whitney U test was applied. For inter-zone comparison, a 1-way ANOVA was used in the case of normal distribution, and a Kruskal–Wallis test with Bonferroni correction for multiple comparison was used in case data that were not normally distributed. Data were presented by GraphPad Prism 10 (Version 10.3.1 GraphPad Software Inc., San Francisco, CA, USA).

## 3. Results

After processing the rabbit Achilles tendons harvested from non-treated contralateral legs (NT group) from experimental groups with a pure DegraPol^®^ tube (Tube-only group) and with a DegraPol^®^ tube and incorporated PDGF-BB growth factor to be released (Tube+GF group), we have stained them with Herovici and with collagen I and III immunohistochemical labeling for comparison of the collagen I-to-III ratio.

### 3.1. Herovici and Immunohistochemistry: Histology

**Figure 1:**
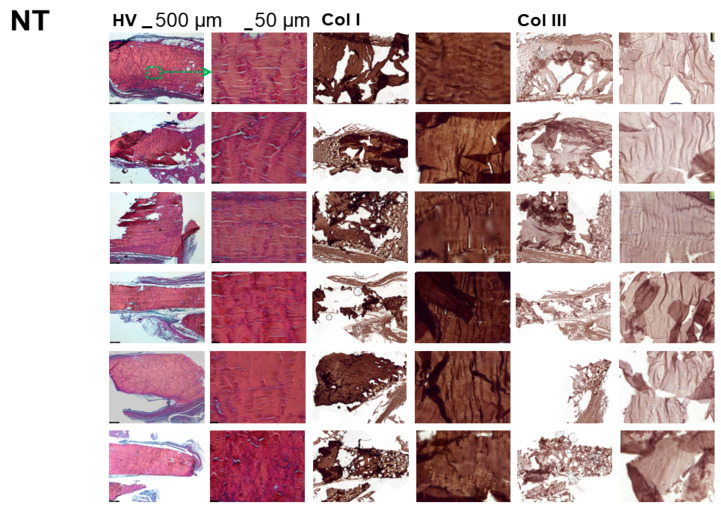
Images taken by brightfield microscopy from rabbit Achilles tendons (AT) of the not-treated group (NT). The first two columns indicate samples that were stained by Herovici (HV), columns 3 and 4 underwent collagen I (Col I) immunohistochemical labeling and columns 5 and 6 underwent collagen III (Col III) immunohistochemical labeling. The green dotted square shows the area that is shown by higher magnification on the subsequent column on the right-hand side. Three rabbit donors were used. Rows 1, 3 and 5 were length sections of the AT in proximity of the bone and rows 2, 4 and 6 represent the length section of the AT in proximity to the muscle.

Tendon tissue is not easily sectioned with the microtome because it is an extremely densely packed connective tissue, resulting in the challenge to achieve and present continuous sections. We here present length sections and for each rabbit (*n* = 3 donors), we show one representative image taken from the tendon tissue in proximity to the muscle and a corresponding one in proximity to the bone. We found that these locations were very similar with regard to the tendon tissue composition in the healing area, as shown in Supplementary Information Figure S1, where the sides were compared for HV, collagen I and collagen III for NZ and SZ, respectively. While Herovici (HV) staining did not impact the 5 μm-thick tendon tissue, the protocol for immunohistochemical labeling required harsh conditions, such as heating the sections up to 95 °C, which resulted in the folding of the tissue (Figure 1).

### 3.2. The Collagen I-to-III Ratio in Different Zones of the Healing Tendon

**Figure 2:**
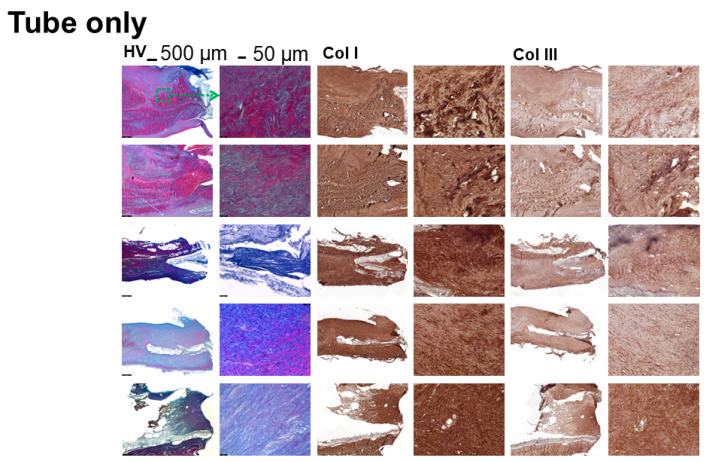
Images taken by brightfield microscopy from rabbit Achilles tendons (AT) of the experimental group with a pure DegraPol^®^ tube set around the transected AT (Tube-only). The first two columns indicate samples that were stained by Herovici (HV), columns 3 and 4 underwent collagen I (Col I) immunohistochemical labeling and columns 5 and 6 underwent collagen III (Col III) immunohistochemical labeling. The green dashed square shows the area that is shown by higher magnification on the subsequent column on the right-hand side. Three rabbit donors were used. Rows 1, 3 and 5 were length sections of the AT in proximity of the bone and rows 2 and 4 represent the length section of the AT in proximity to the muscle (the tendon tissue in proximity of the bone of donor 3 is missing).

**Figure 3:**
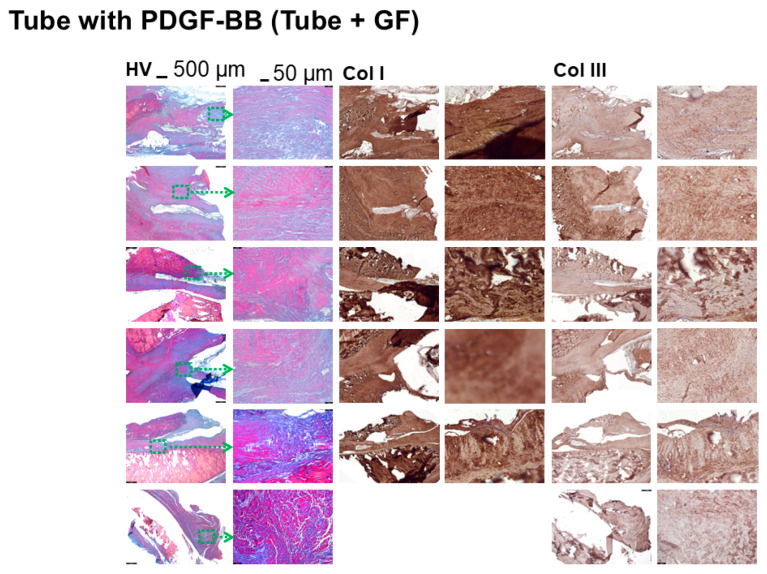
Images taken by brightfield microscopy from rabbit Achilles tendons (AT) of the experimental group with a pure DegraPol^®^ tube with incorporated PDGF-BB set around the transected AT (Tube+GF). The first two columns indicate samples that were stained by Herovici (HV), columns 3 and 4 underwent collagen I (Col I) immunohistochemical labeling and columns 5 and 6 underwent collagen III (Col III) immunohistochemical labeling. The green dashed square shows the area that is shown by higher magnification on the subsequent column on the right-hand side. Three rabbit donors were used. Rows 1, 3 and 5 were length sections of the AT in proximity of the bone and rows 2, 4 and 6 represent the length section of the AT in proximity to the muscle. The collagen I IHC labeling for donor 3 and tendon tissue in proximity to the muscle is missing.

**Figure 4:**
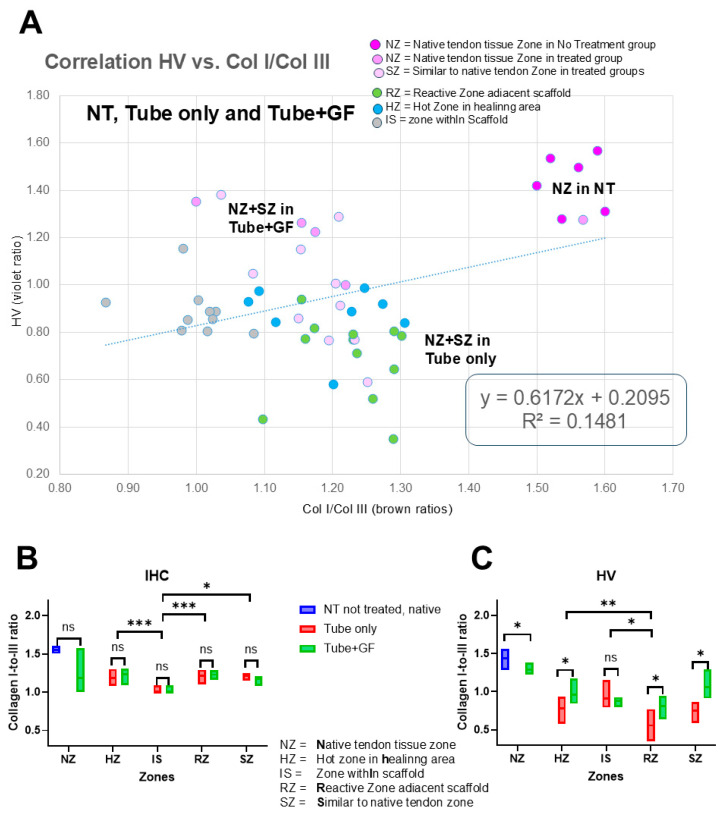
Collagen I-to-III ratio assessed by HV (*y*-axis) and by IHC (*x*-axis) for different zones of the healing tendon for all three experimental groups. The zones are depicted with different-colored dots and explained in the upper part of the Figure, on the right side. Linear regression analysis with the slope and correlation coefficient is shown in the square on the lower right side (**A**). The experimental groups were compared for each zone for IHC (**B**) and for HV (**C**), and inter-group comparison was statistically calculated using a non-parametric Mann–Whitney U test for two independent groups. For the inter-zone comparison, a Kruskal–Wallis test was performed with adjustment by a Bonferroni correction of *p* values. Non-parametric tests were utilized because data were not normally distributed. Note: As the NT group of the NZ zone showed a significant difference to all other zones, with significantly higher values, we have omitted the corresponding lines/asterisks for better visibility. Key: ns = not significant; *p* < 0.05 (*); *p* < 0.01 (**) and *p* < 0.001 (***).

Using Figure 1, Figure 2 and Figure 3, we assessed the collagen I-to-III ratio in five FOVs of four different zones for each section and compared the results obtained from the HV-stained sections with the ones based on IHC brown intensities. An overview of the collagen I-to-III ratios for the three experimental groups is shown in Figure 4. Several distinct zones were analyzed. NZ represents the zones of native tendon tissues in NT and treated groups, i.e., the Tube-only and Tube+GF groups. SZ represent the zone of native tendon-looking tissue, which was near the injury site; SZ is an abbreviation for “similar” zone. Furthermore, the zone immediately adjacent to the implanted material was called RZ, indicating the reactive zone. HZ was used for the healing tendon tissue zone (hot zone), meaning the area in the core tendon tissue that was undergoing healing processes. Finally, as tissue had also integrated into the scaffold implant material, we defined the last zone as IS, representing the tissue within the scaffold (IS = in the scaffold, tissue between the fibers of the scaffold). Figure 4 shows a weak positive correlation between the two staining techniques that were used to assess the collagen I-to-III ratio. Furthermore, it clearly shows clusters where data accumulate that were determined and calculated for different zones, with the most prominent example of the NT group showing by far the highest collagen I-to-III ratio. In addition, we compared experimental groups for each zone assessed by IHC (Figure 4B) and by HV (Figure 4C). While inter-group comparison based on IHC only revealed small differences that were not significant, HV showed significant differences in collagen I-to-III ratios, when Tube+GF was compared to the Tube-only group. Specifically, in HZ, RZ and SZ, the ratio was significantly higher in Tube+GF-treated tendons. When the NZ zone of NT tendons that were not treated (native tendons from contralateral side) was compared to the NZ zone from GF-treated tendons, the collagen I-to-III ratio was significantly higher for the NT tendons. Inter-zone comparison revealed that the ratios were significantly smaller for IS compared to HZ, RZ and SZ, based on IHC data analysis. For the HV-based analysis, RZ values for the collagen I-to-III ratios were smaller than found in IS and in HZ, respectively. Hence, HV-based analysis showed a greater dynamic range and revealed higher discrimination between experimental groups and anatomical zones than IHC did, which caused the weak positive correlation between the two methods illustrated in Figure 4.

**Figure 5:**
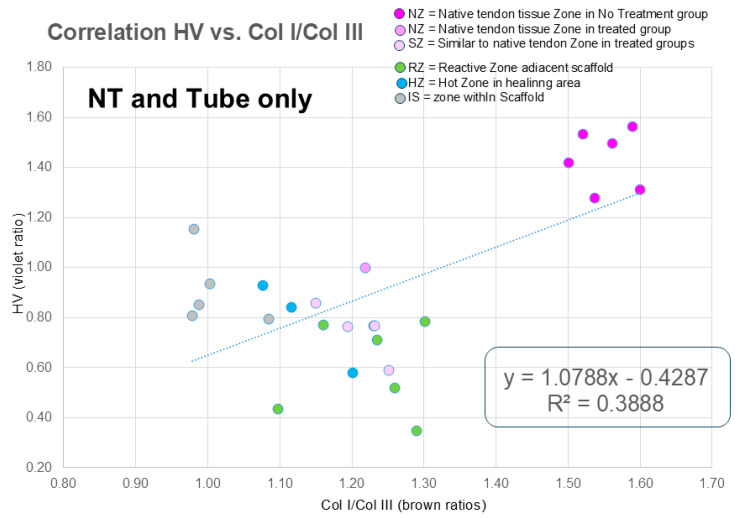
Collagen I-to-III ratio assessed by HV (*y*-axis) and by IHC (*x*-axis) for different zones of the healing tendon for the NT group and Tube-only group. The zones are depicted with different-colored dots and explained in the upper part of the Figure, on the right side. Linear regression analysis with the slope and correlation coefficient are shown in the square on the lower right side.

**Figure 6:**
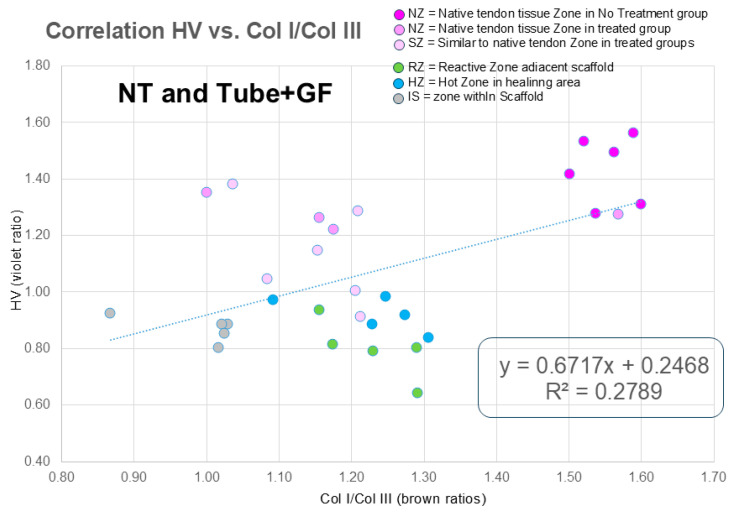
Collagen I-to-III ratio assessed by HV (*y*-axis) and by IHC (*x*-axis) for different zones of the healing tendon for the NT group and Tube+GF group. The zones are depicted with different-colored dots and explained in the upper part of the Figure, on the right side. Linear regression analysis with the slope and correlation coefficient are shown in the square on the lower right side.

For better visibility, Figure 5 and Figure 6 show the same correlation data; however, this is only with two experimental groups: Figure 5 shows data from the NT and Tube-only groups, while Figure 6 shows data from the NT and Tube+GF groups, respectively. The correlation coefficients slightly improved compared to Figure 4, where the data of all groups were plotted, indicating that the correlation was dependent on the groups that were analyzed.

## 4. Discussion

The surgical treatment of ruptured tendons necessitates implant materials that alleviate the two main problems during tendon healing: namely, re-rupture caused by inferior mechanical strength and fibrotic adhesion to the surrounding tissue [17,18]. Innovative strategies, including targeted drug delivery vehicles for local therapy, have emerged [19]. To treat healing tendons and support a more regenerative healing, rather than fibrosis, novel medical devices using innovative (bio) scaffolds and combining different cell sources, biodegradable materials and immune modulators have been reviewed recently [20]. Specifically, synthetic, natural or composite scaffold materials with different degradation rates have been explored [21]. Furthermore, gene delivery was tested to support healing [22]. Another approach is the delivery of growth factors to the healing tendon, such as insulin-like growth factor-1 (IGF-1) [23], transforming growth factor beta (TGF-*β*) [24], vascular endothelial growth factor (VEGF) [25], platelet-derived growth factor (PDGF) [26], bone morphogenetic proteins (BMPs) [27] and basic fibroblast growth factor (bFGF) [28], respectively. Previously, we also used PDGF-BB to stimulate tendon healing. Already, three weeks post-operation, a significantly higher load-until-failure of the PDGF-BB tube-treated tendons was determined, with double the strength compared to a pure DegraPol^®^ tube without PDGF-BB [10]. We assumed that PDGF-BB accelerated tendon healing in this experiment, because such high load-until-failure values are only expected to occur at later time points during the healing process [18].

To examine the healing status of tendons, besides the cell density, particularly of Scleraxis positive cells [29] or myofibroblasts [30], and the extent of vascularization [31], the collagen I-to-III ratio can be used as a proxy [32], because collagen III is deposited first and is only gradually replaced by type I collagen later [33]. We therefore took the opportunity to compare classic immunohistochemical labeling for collagen I and III, with more recently developed dual color Herovici staining methods to assess the collagen I-to-III ratio in different zones of the healing tendons under these different therapeutic conditions, with and without PDGF-BB delivery from a coaxially electrospun tube implanted around a fully transected and sutured rabbit Achilles tendon.

The major findings were that native tendons had the highest collagen I-to-III ratios, while the zones within the scaffold fibers and adjacent to the implant exhibited the lowest collagen I-to-III ratios (Figure 4 and Supplementary Information Figures S2–S8). The zones in the tendon core, distal to the implant, showed higher ratios compared to the zone within the scaffold and in immediate proximity to the implant. Both staining methods yielded roughly similar results; however, the HV method showed a higher dynamic range and better discrimination between specific zones. Also, HV yielded significant differences between the Tube-only and Tube+GF groups for HZ, SZ and RZ, respectively, while the differences were smaller and not significant for the IHC-based collagen I-to-III ratios (though still recognizable as trends). Regarding the therapeutic aspect, we found that, indeed, the PDGF-BB treatment resulted in higher collagen I-to-III ratios for similar-to-native tendon tissue zones when compared to the tube without PDGF-BB treatment. In accordance, the “hot” zone in the healing area showed a significant shift to higher collagen I-to-III ratios under PDGF-BB treatment (Supplementary Information Figure S4).

The first hypothesis of this study was that HV and IHC-based collagen I-to-III ratios are similar for the different zones and experimental groups that we used. However, the correlation for HV readouts versus IHC readouts was only weakly positive, with a slope of 0.62 and an R^2^ of only 0.15 (Figure 4A). The correlation coefficient improved when only NT and Tube+GF groups were used for the correlation (Figure 6; R^2^ = 0.28) or only NT and Tube-only groups (Figure 5; R^2^ = 0.39). Separate analyses of IHC and HV, along with statistical comparisons of the experimental groups for each zone (Figure 4B,C), revealed a significant increase in the collagen I-to-III ratio following PDGF-BB treatment in the HV analysis. This is consistent with the literature, which reports that increased levels of collagen type III relative to type I predispose tendons to rupture, as collagen III possesses lower mechanical strength than type I [34] and therefore can at least partly explain the higher load-until-failure we had discovered for PDGF-BB treatment earlier [10]. While the NZ and SZ values were clearly distinguishable from the HZ values and even more so from the RZ values in the correlation plots and using the HV staining method, the NZ, SZ, HZ and RZ were less distinguishable in the ratios that were determined using IHC-labeled sections (Figure 4A, Figure 5 and Figure 6). Although the native (not treated) tendon tissue of the contralateral legs’ collagen I-to-III ratio values was clearly clustered for both staining methods in the upper right corner of the correlation plot (Figure 4A), with the highest collagen I-to-III ratios, the more subtle differences found between NZ, SZ, HZ and RZ yielded a clearer allocation spectrum in the HV-stained, rather than IHC-labeled sections, because the data exhibited a larger range for these zones (Supplementary Information Figures S2–S5), which was confirmed by the separate and quantitative analysis of IHC and HV data (Figure 4B,C).

Regarding the second hypothesis, PDGF-BB treatment led to a shift from similar-to-native tendon tissue zones (SZ) to higher collagen I-to-III ratios, which was confirmed by quantitative HV-based analysis (Figure 4C and Supplementary Information Figure S2). In the healing zone (HZ), PDGF-BB significantly increased the collagen I-to-III ratio when assessed by HV, though IHC analysis showed no significant difference (Supplementary Information Figure S4 and Figure 4B,C). Similarly, increased ratios were found for the reactive zone near the implant (RZ) under PDBF-BB treatment (Supplementary Information Figure S3 and Figure 4C), while for the tendon tissue that was deposited by tenocytes within the fiber mesh of the tubes (IS), the overall rather low collagen I-to-III ratios were similar for the two treatment groups, i.e., for Tube-only and Tube+GF, respectively (Supplementary Information Figure S5 and Figure 4B,C). Consequently, our hypothesis was confirmed for the SZ, HZ and RZ zones, but not for the intraspun IS zone.

The final hypothesis proposed that proximity to the PDGF-BB-releasing fibers would correlate with higher collagen I-to-III ratios, expecting a decline in the order IS > RZ > HZ > SZ in PDGF-BB-treated groups. However, our data revealed that collagen deposition within the implant (intraspun IS zone) showed rather low values, suggesting that the maturation from collagen III to collagen I was retarded and relatively slow within the electrospun fiber pores. Because tenocytes have to migrate into the scaffold pores and to deposit collagen there, this finding seems reasonable (it takes more time for these cells to reach the pores than the injury site in the core), despite the expected higher local PDGF-BB concentrations close to the fibers within the implant that would stimulate the tenocytes to produce collagen [26,35].

The intraspun IS zone and the reactive zone at the interface to the implant (RZ) exhibited rather low collagen I-to-III ratios despite the probable highest local PDGF-BB concentration being caused by proximity to the bioactive fibers. As a matter of fact, the observed order turned out to be SZ > HZ> IS ~ RZ (Figure 4), which is opposed to our expectation. These findings suggest that locally elevated PDGF-BB concentrations do not automatically result in an advanced healing status in those specific areas. Instead, tenocyte migration into these zones appears to be the rate-limiting factor. Nevertheless, PDGF-BB delivery promotes a more advanced healing state across specific anatomical zones, with the most pronounced effects observed in the healing core (HZ) and the similar-to-native (SZ) zones.

During acute tendon injury, intrinsic healing by tissue resident tenocytes and extrinsic healing by tenocytes from the peritenon and surroundings are both needed for the repair of the lacerated site, although the predominance of extrinsic fibroblasts and tenocytes are associated with adhesion formation [36]. In our tube-treated tendons, extrinsic healing is suppressed by the placement of the tube around the sutured site (barrier function). Therefore, data obtained for the collagen I-to-III ratio are attributed to a predominant intrinsic healing by the resident tenoblasts and tenocytes responsible for collagen III deposition [37] and, later, for collagen I deposition. Such tendon tissue resident tenocytes have to migrate to the different zones we have defined in this study, which needs more time in case the zone is within the scaffold material (IS), rather than in the core healing zone (HZ), respectively. On the other hand, the growth factor must diffuse through the tendon tissue to reach the core healing zone, and its concentration is probably highest in the IS and in the reactive zone (RZ) that is adjacent to the implant. The diffusion coefficient of PDGF-BB (24.5 kDa) in aqueous solution is approximately 10^−6^ cm^2^s^−1^ [38]; however, it is estimated to be 10 to 100 times lower in dense connective tissues such as tendons. In vivo fluorescence studies using bovine serum albumin (BSA, 66 kDa) as a model protein have demonstrated diffusion distances reaching 3.5 mm from the implant site within three weeks [38]. Given that the tendon injury site likely exhibits low density—comprising loose, disorganized fibers with a high water content—similar diffusion distances are expected in our model. Since the rabbit Achilles tendon has a diameter of approximately 3.5 to 4 mm, PDGF-BB should effectively diffuse throughout the injury site within three weeks to stimulate the resident tendon cells. Hence, both the availability and migration capacity of the resident tendon cells, on the one hand, as well as the diffusion rate and distribution dynamics of the growth factor to different zones of the healing tissue, on the other hand, are supposed to influence the local healing status simultaneously. This interplay results in a heterogeneous healing state throughout the tendon, with the degree of maturation varying significantly, depending on the specific zone.

This study has several limitations. First, the sample size was limited to three rabbits per experimental group. While this was necessitated by high experimental costs, it aligns with the 3Rs principle of animal welfare by minimizing the number of animals used [39]. Second, the study focused on a single time point at three weeks post-operation. This specific endpoint was selected to allow for a direct comparison with and a more in-depth interpretation of the previous findings, which demonstrated a doubling of failure strength in the rabbit Achilles tendon model under local PDGF-BB delivery [10]. Finally, a technical limitation of the IHC-based collagen I-to-III ratio calculation involves the potential for tissue folding during antigen retrieval; such artifacts can interfere with the accuracy of the red-to-green intensity assessment.

## 5. Conclusions

We provide local collagen I-to-III ratios for healing rabbit Achilles tendons after full transection and conventional suture, three weeks post-operation, in different zones of the tendon and assessed by two different staining methods. While HV and IHC were weakly positively correlated for the assessed ratios, HV provided a larger dynamic range of values and was more robust in this specific model and workflow. Based on HV analysis, the PDGF-BB therapy induced significantly higher collagen I-to-III ratios in most of the analyzed zones, except for the intraspun zone (IS). This was explained by the fact that the resident tendon cells had to migrate into the fibers in both treatment groups, so that even the high local PDGF-BB concentrations that are assumed for pores within the fiber mesh did not lead to the expected maturation effect in IS. These findings suggest that Herovici staining may be the more robust choice for determining the collagen I-to-III ratio in this animal model. Furthermore, our data indicate that a drug-releasing tube does not necessarily accelerate healing in its immediate proximity or interior; instead, the advanced state of healing was more evident in regions that were distal to the implant material, although it has to be emphasized that this observation is restricted to this specific model and a time point of 3 weeks post-surgery. Nevertheless, the finding of lower maturity of the collagens despite the proximity to PDGF-BB-releasing fibers can be judged as a strength of this study. Consequently, healing tissues should not only be analyzed at different time points, but also with a spatial resolution for different zones in the future, particularly if drug-delivery vehicles are used.

## Data Availability

Data are contained within the article.

## Supplementary Materials

The following supporting information can be downloaded at: https://www.mdpi.com/article/10.3390/biology15020204/s1, Figure S1: Comparison of red-to-green and red-to-blue ratios of HV-stained sections for muscle side (Mu), bone side (Bo) and average of both (Mu + Bo) for NZ and SZ and the three experimental groups (DP = Tube-only; DP + GF = Tube with PDGF-BB; and NT = not-treated contralateral legs, native tendon tissue). For collagen I (Col 1) and collagen III (Col 3), the red-to-green and blue-to-red ratios are given, again for the comparison of Mu, Bo and Mu + Bo, respectively. Mean and standard deviation are shown. Figure S2: Collagen I-to-III ratio assessed by HV (*y*-axis) and by IHC (*x*-axis) for different zones of the healing tendon for the two experimental groups. The zones are depicted with different-colored dots and explained in the upper part of the Figure, on the right side. Figure S3: Collagen I-to-III ratio assessed by HV (*y*-axis) and by IHC (*x*-axis) for different zones of the healing tendon for the two experimental groups. The zones are depicted with different-colored dots and explained in the upper part of the Figure, on the right side. Figure S4: Collagen I-to-III ratio assessed by HV (*y*-axis) and by IHC (*x*-axis) for different zones of the healing tendon for the two experimental groups. The zones are depicted with different-colored dots and explained in the upper part of the Figure, on the right side. Figure S5: Collagen I-to-III ratio assessed by HV (*y*-axis) and by IHC (*x*-axis) for different zones of the healing tendon for the two experimental groups. The zones are depicted with different-colored dots and explained in the upper part of the Figure, on the right side. Figure S6: Five fields of view (FOVs) per section towards the bone site and towards the muscle site for 3 native (not-treated contralateral) tendons (NT); stained with Herovici (HV) or labeled for collagen I (Col I) or collagen III (Col III) for the NZ zone = native tendon zone. Figure S7: Five fields of view (FOVs) in four different zones per section towards the bone site and towards the muscle site for 3 tube-only-treated tendons, meaning tube application without PDGF-BB (Tube-only); stained with Herovici (HV) or labeled for collagen I (Col I) or collagen III (Col III) in the healing tendon tissue, explained in the Figure at the bottom. Figure S8: Five fields of view (FOVs) in four different zones per section towards the bone site and towards the muscle site for 3 tendons receiving PDGF-BB, meaning tube application with the growth factor PDGF-BB (Tube+GF); stained with Herovici (HV) or labeled for collagen I (Col I) or collagen III (Col III) in the healing tendon tissue, explained in the Figure at the bottom.
