# Supplementary material for: Therapeutically Induced Modulation of Collagen I-to-III Ratio Three Weeks After Rabbit Achilles Tendon Full Transection
Source: Biology (Basel). 2026 Jan 22;15(2):204. doi: 10.3390/biology15020204 (PMC12837842; doi:10.3390/biology15020204)
Supplement: Supplementary file 1 [file biology-15-00204-s001.zip › biology-4078058-supplementary.pdf]

Supporting Information

Therapeutically Induced Modulation of Collagen I-to-III Ratio Three Weeks After Rabbit Achilles Tendon Full Transection

Gabriella Meier Bürgisser, Olivera Evrova, Pietro Giovanoli, Maurizio Calcagni and Johanna Buschmann *

Department of Plastic Surgery and Hand Surgery, University Hospital Zurich, 8091 Zurich, Switzerland; gabriella.meierbuergisser@usz.ch (G.M.B.); olivera.evrova@gmail.com (O.E.); pietro.giovanoli@usz.ch (P.G.); maurizio.calcagni@usz.ch (M.C.)

* Correspondence: johanna.buschmann@usz.ch; Tel.: +41-44-255-98-95

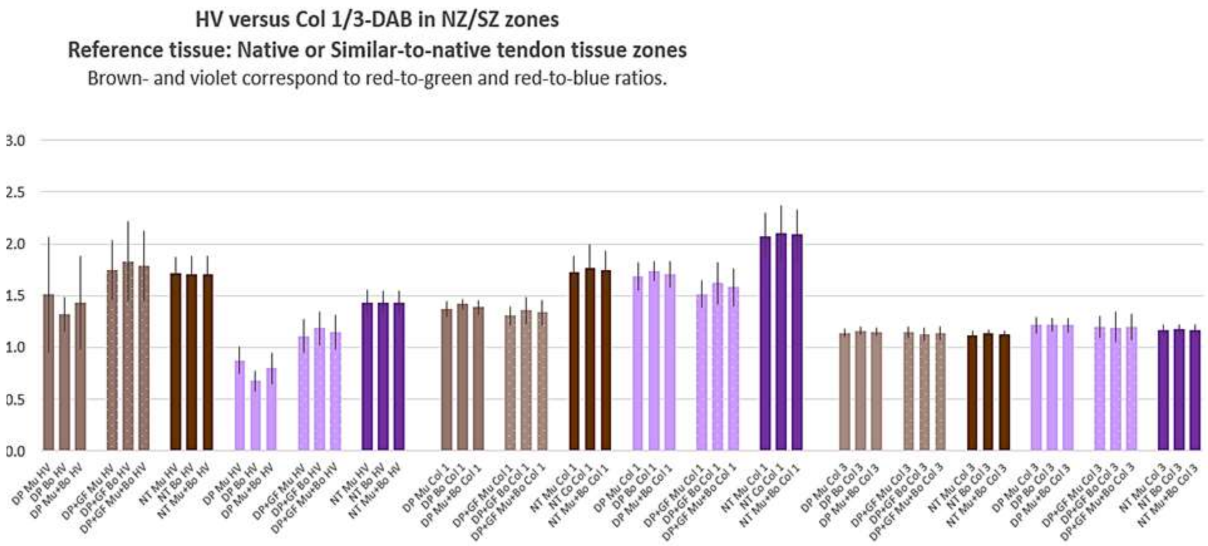

SI Figure S1 Comparison of red-to-green and red-to-blue ratios of HV stained sections for Muscle side (Mu), Bone side (Bo) and average of both (Mu + Bo) for NZ and SZ and the three experimental groups (DP = Tube only; DP + GF = Tube with PDGF-BB; and NT = not treated contralateral legs, native tendon tissue). For collagen I (Col 1) and collagen III (Col 3), the red-to-green and blue-to-red ratios are given, again for the comparison of Mu, Bo and Mu + Bo, respectively. Mean and standard deviation are shown.

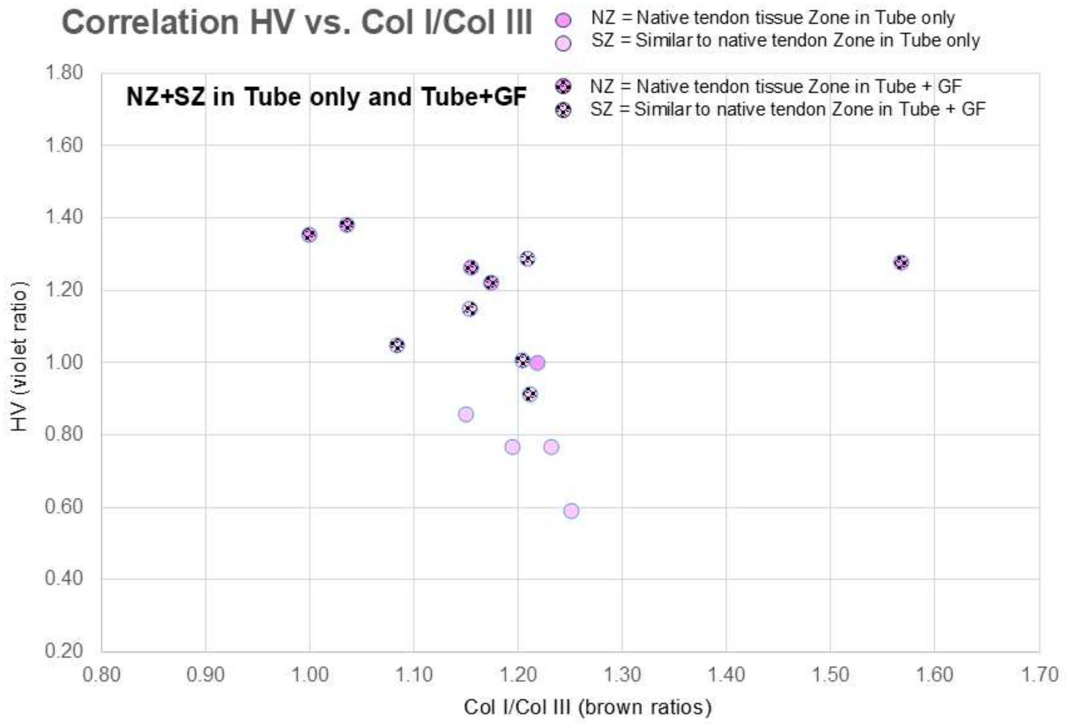

SI Figure S2 Collagen I-to-III ratio assessed by HV (y-axis) and by IHC (x-axis) for different zones of the healing tendon for the two experimental groups. The zones are depicted with differently colored dots and explained in the upper part of the Figure on the right side.

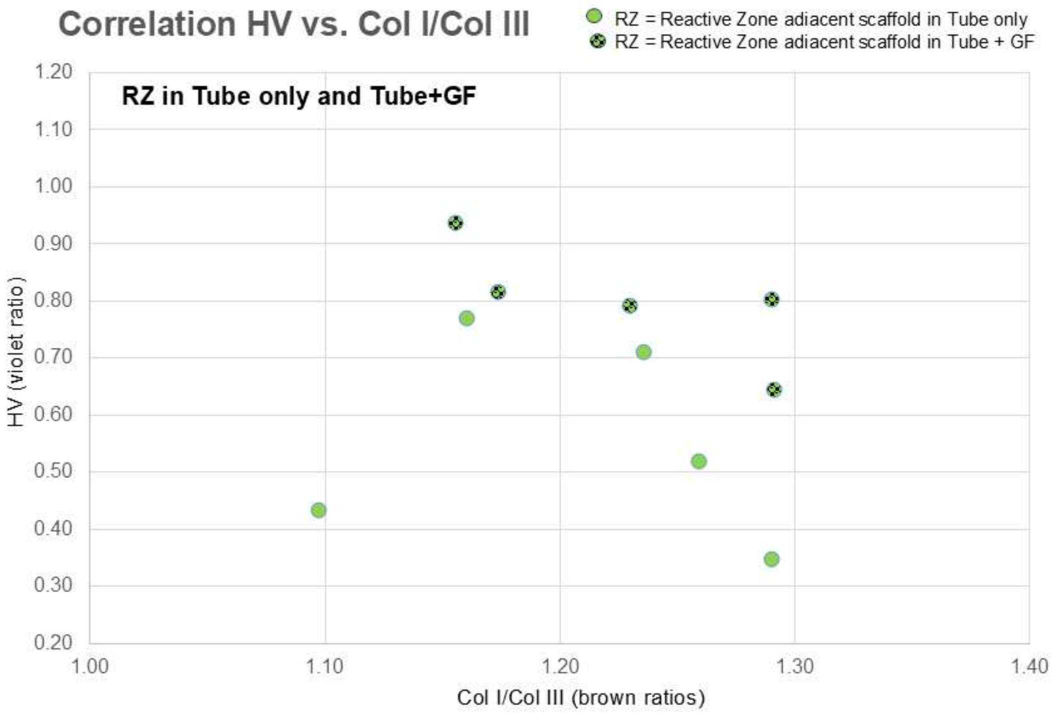

SI Figure S3 Collagen I-to-III ratio assessed by HV (y-axis) and by IHC (x-axis) for different zones of the healing tendon for the two experimental groups. The zones are depicted with differently colored dots and explained in the upper part of the Figure on the right side.

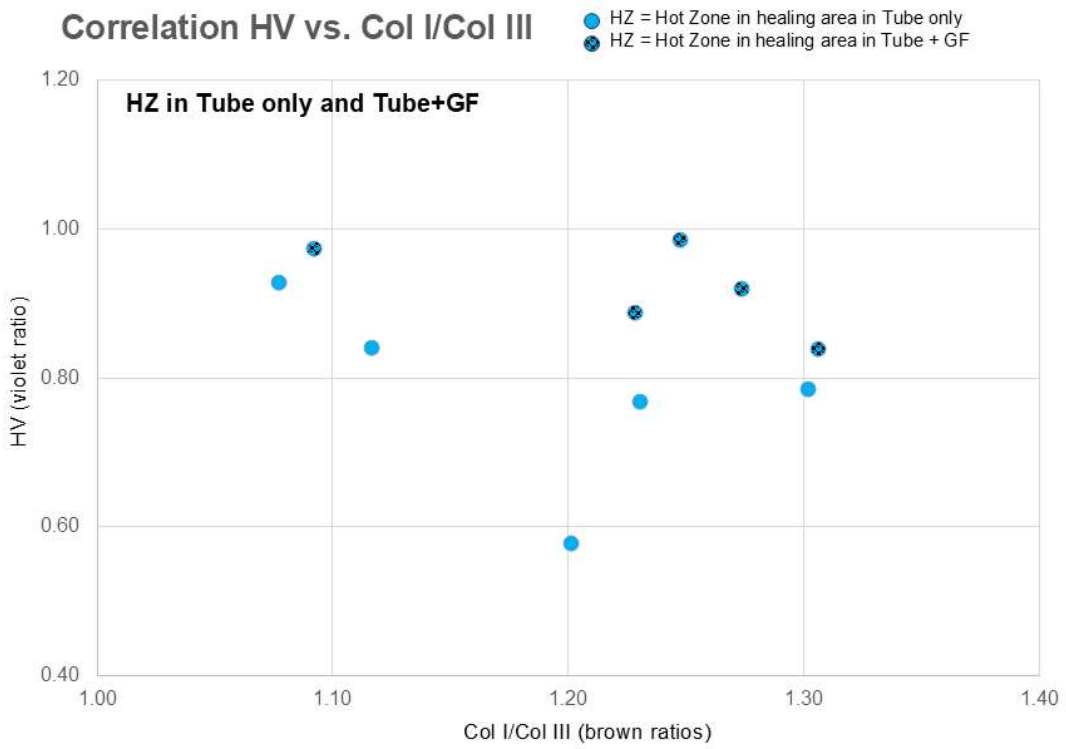

SI Figure S4 Collagen I-to-III ratio assessed by HV (y-axis) and by IHC (x-axis) for different zones of the healing tendon for the two experimental groups. The zones are depicted with differently colored dots and explained in the upper part of the Figure on the right side.

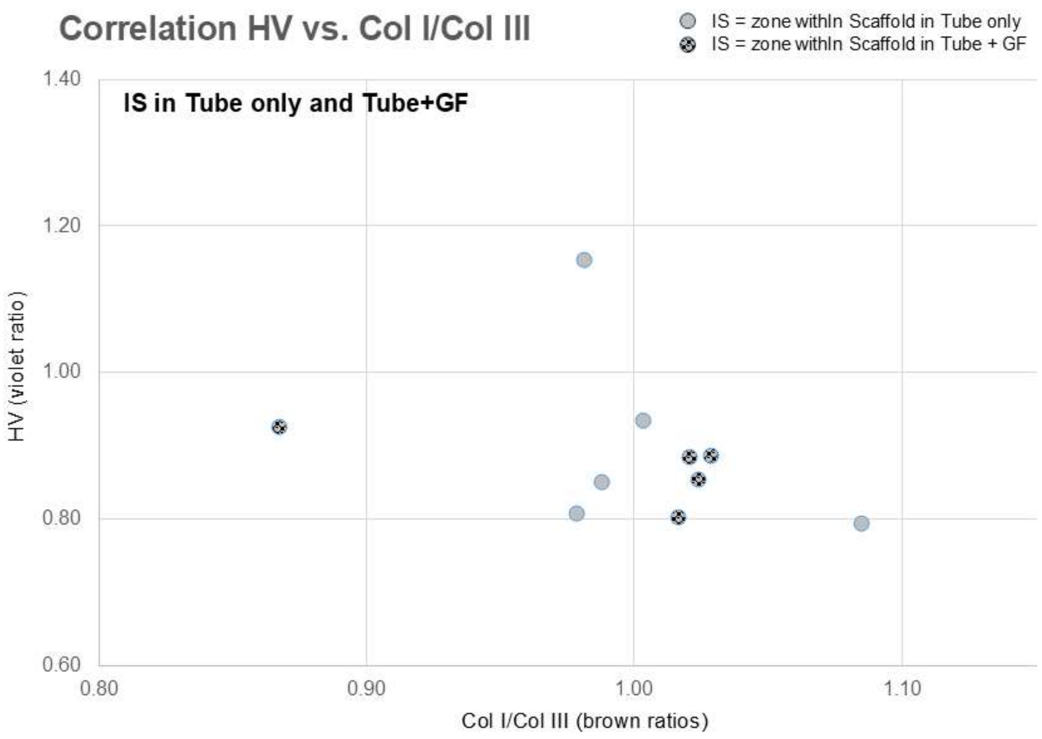

SI Figure S5 Collagen I-to-III ratio assessed by HV (y-axis) and by IHC (x-axis) for different zones of the healing tendon for the two experimental groups. The zones are depicted with differently colored dots and explained in the upper part of the Figure on the right side.

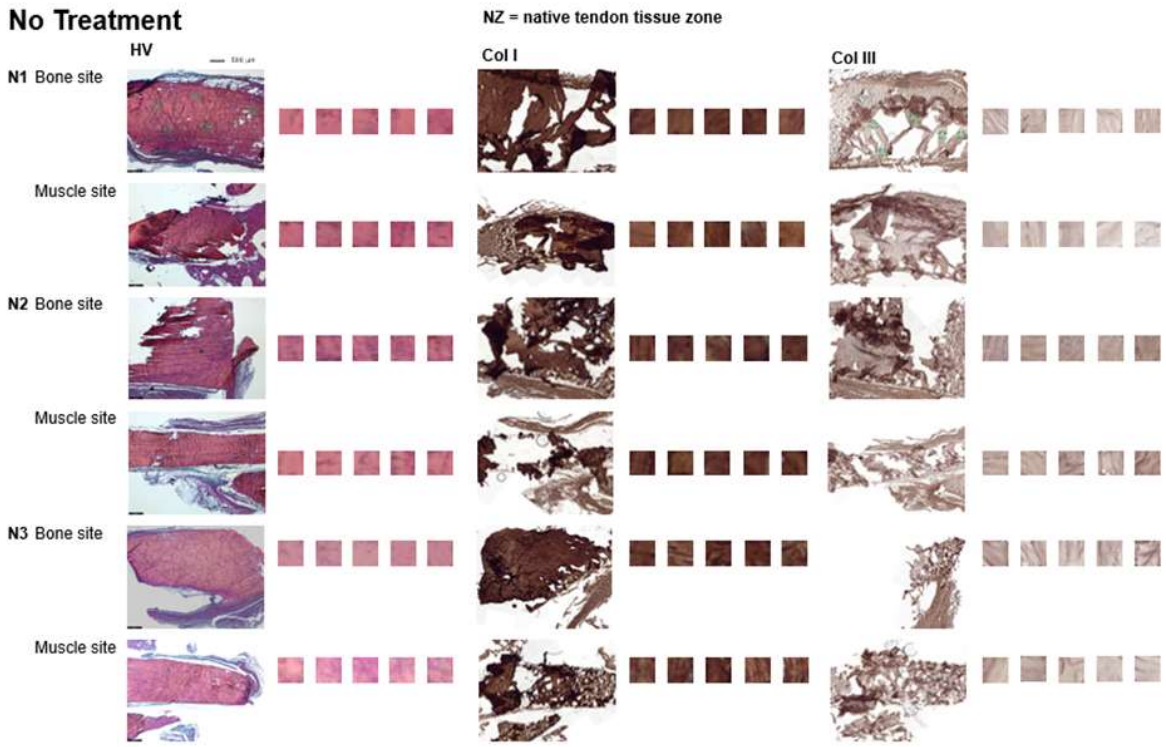

SI Figure S6 Five fields of view (FOVs) per section towards the bone site and towards the muscle site for 3 native (not treated contralateral) tendons (NT); stained with Herovici (HV) or labeled for Collagen I (Col I) or Collagen III (Col III) for the NZ zone = native tendon zone.

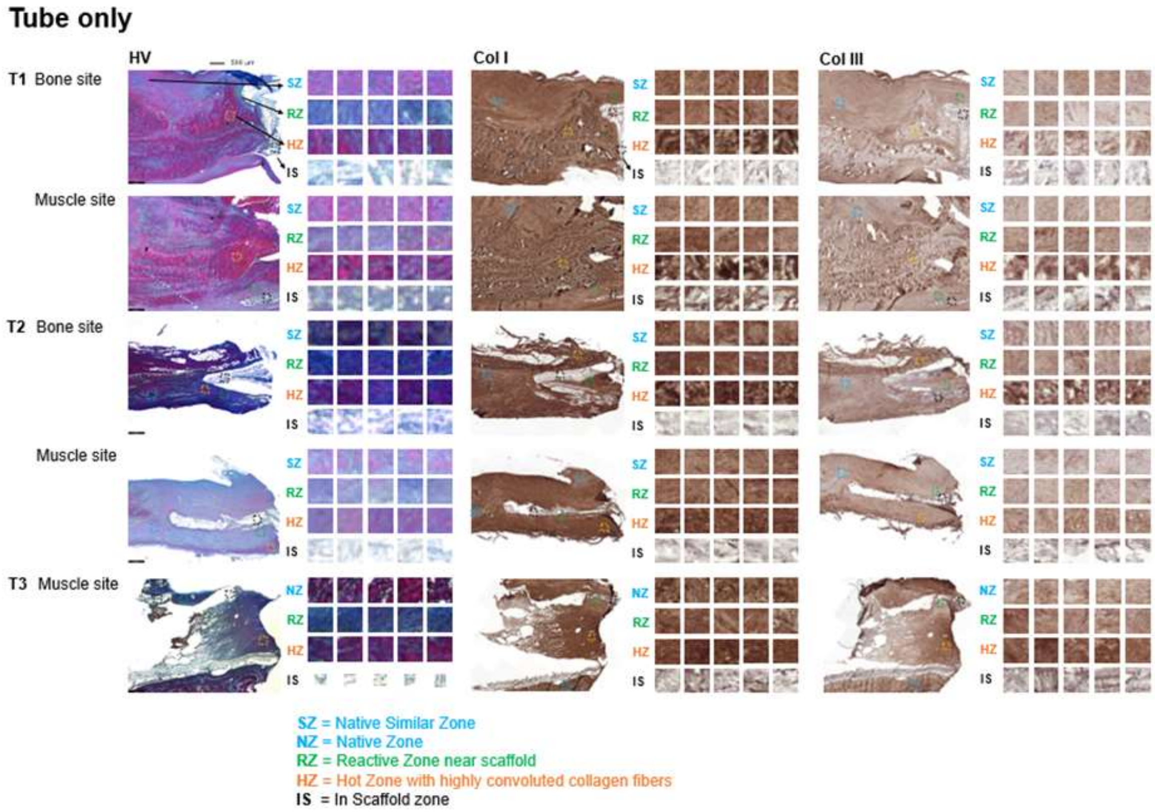

SI Figure S7 Five fields of view (FOVs) in four different zones per section towards the bone site and towards the muscle site for 3 tube only treated tendons, meaning tube application without PDGF-BB (Tube only); stained with Herovici (HV) or labeled for Collagen I (Col I) or Collagen III (Col III) in the healing tendon tissue, explained in the Figure at the bottom.

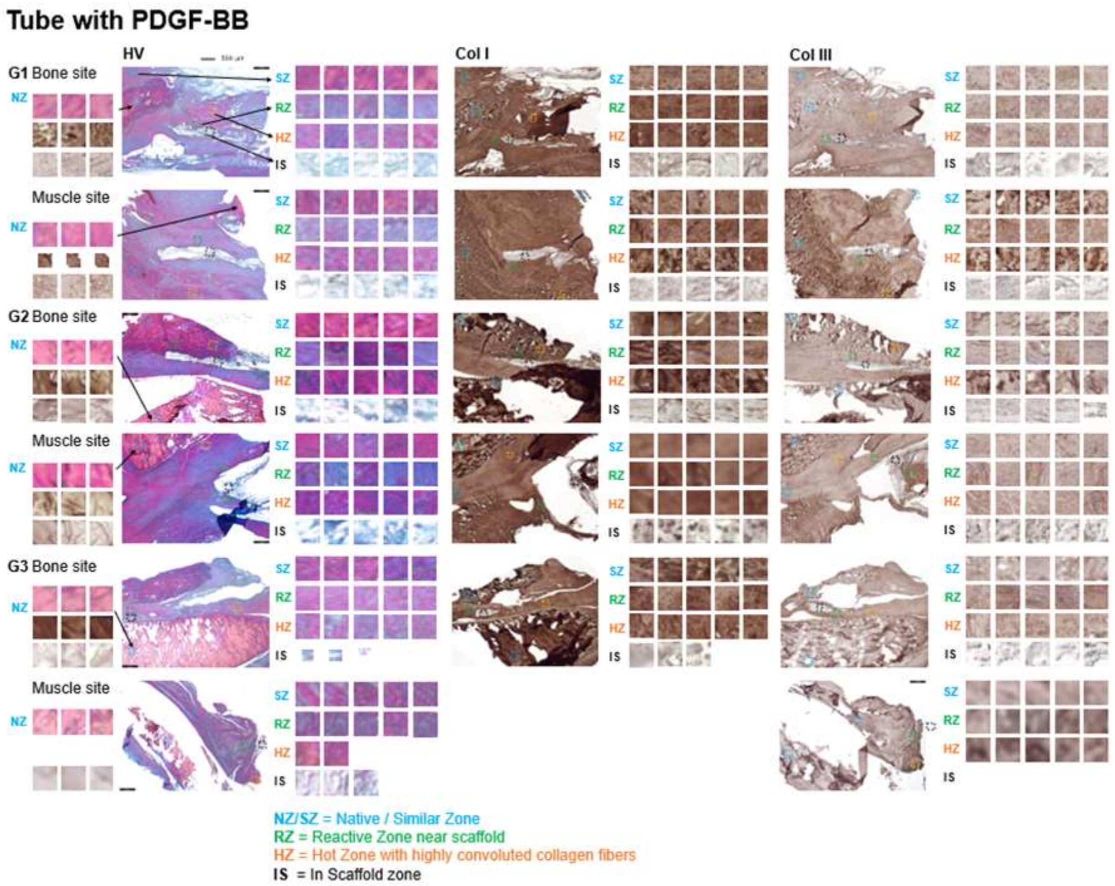

SI Figure S8 Five fields of view (FOVs in four (five) different zones) per section towards the bone site and towards the muscle site for 3 tendons receiving PDGF-BB, meaning tube application with the growth factor PDGF-BB (Tube+GF); stained with Herovici (HV) or labeled for Collagen I (Col I) or Collagen III (Col III) in the healing tendon tissue, explained in the Figure at the bottom.
